# Identification of 9-Core Immune-Related Genes in Bladder Urothelial Carcinoma Prognosis

**DOI:** 10.3389/fonc.2020.01142

**Published:** 2020-07-08

**Authors:** Lei Na, Yu Bai, Yu Sun, Zhuo Wang, Wei Wang, Lin Yuan, Chenghai Zhao

**Affiliations:** ^1^Department of Pathophysiology, College of Basic Medical Science, China Medical University, Shenyang, China; ^2^Department of Urology, Shengjing Hospital of China Medical University, Shenyang, China; ^3^Department of Nephrology, Shengjing Hospital of China Medical University, Shenyang, China; ^4^Liaoning Branch of China Telecom, Shenyang, China

**Keywords:** immune, bladder urothelial carcinoma (bladder cancer), prognosis, overall survival, TCGA

## Abstract

**Background:** Immune microenvironment within tumors affects initiation, progression and clinical outcome of human cancers. Here we explored an immune-related gene signature associated with prognosis of patients with bladder urothelial carcinoma.

**Method:** The Cancer Genome Atlas (TCGA) database was interrogated for expressions of immune-related genes in bladder urothelial carcinomas. Integrated bioinformatics analyses were performed to identify prognostic factors.

**Results:** Twenty-seven immune-related genes were revealed significantly associated with patient's overall survival (OS) by univariate Cox proportional hazards regression analysis. Nine-core immune-related genes including *MMP9, PDGFRA, AHNAK, OLR1, RAC3, IGF1, PGF, OAS1*, and *SH3BP2* were selected to construct a risk score model by multivariate Cox proportional hazards regression analysis. Bioinformatics analyses further validated that risk score could be used as an important independent factor in evaluating prognosis.

**Conclusion:** We established a prognostic immune signature for patients with bladder urothelial carcinoma, which may provide novel targets for prediction and therapy of these patients.

## Introduction

Bladder cancer incidence ranks first in men among malignant cancers of the urinary system in China and second only to prostate cancer in Western countries (1). In the USA, according to the latest data, there will be 80,470 new cases and 17,670 deaths in 2019 (2). The incidence of bladder cancer in men is three to four times higher than that in women (2). Bladder cancer consists of two major types, non-muscle-invasive bladder cancer (NMIBC) and muscle-invasive bladder cancer (MIBC). NMIBC is associated with a longer survival compared with MIBC, although NMIBC is prone to local recurrence and can develop invasive disease. In contrast, MIBC is usually accompanied by metastasis and unfavorable prognosis. There are no advances in the treatment of metastatic bladder cancer for decades (3, 4). Until recent years, immune checkpoint blockade has been demonstrated to be a promising method and approved for clinical treatment for metastatic bladder cancer (4, 5) (KEYNOTE 045, KEYNOTE 052, IMVIGOR211, IMVIGOR 210).

Bladder cancer is one of the most immunogenic cancers (6, 7). However, bladder cancer can still establish an immunosuppressive microenvironment thereby evading immune surveillance. Bladder cancer is infiltrated by FOXP3^+^ T-cells (Tregs) with production of T_H_1 inhibitory cytokines (8). Moreover, high FOXP3/CD8 ratio is associated with poor prognosis of patients with bladder cancer (9, 10). Bladder cancer is also characterized by infiltration of innate immune cells (11–13). Interaction between tumor cells and immune cells on one hand suppresses immune response, on the other hand promotes tumor progression.

As immune microenvironment affects tumor progression, in the present study, we intended to explore an immune-related gene signature associated with prognosis of patients with bladder urothelial carcinoma, the most common type of bladder cancer. We identified a panel of 9-core differentially expressed genes (DEG) including *MMP9, PDGFRA, AHNAK, OLR1, RAC3, IGF1, PGF, OAS1*, and *SH3BP2*. Using these 9-core DEG, we set up a model to evaluate prognosis of patients with bladder urothelial carcinoma.

## Materials and Methods

### Data Acquisition

A total of 411 urothelial carcinoma samples and 19 matched cancer-adjacent normal samples from TCGA database were included in this study. The transcriptome profiling of RNA expression was corrected by FPKM in TCGA. All data were integrated into one matrix file using a script of Perl language. Ensembl database was used to transform gene IDs into gene symbols. Clinical information was also extract from TCGA (Table 1). Two thousand four hundred ninety-eight immune-related genes listed in the immunology Database and Analysis Portal (ImmPort) were investigated. Moreover, 318 transcription factor-related genes in Critrome were analyzed.

**Table 1:** Clinical features of patients with bladder urothelial carcinoma in TCGA.

**Variable**	**Count (*n* = 405)**
**Status**	
Alive	249 (61.48%)
Dead	156 (38.52%)
**Age**	
≤65	160 (39.51%)
>65	245 (60.49%)
**Gender**	
Female	105 (25.93%)
Male	300 (74.07%)
**Grade**	
Low Grade	21 (5.19%)
High Grade	381 (94.07%)
Missing	3 (0.74%)
**Stage**	
I	2 (0.49%)
II	129 (31.85%)
III	138 (34.08%)
IV	134 (33.09%)
Missing	2 (0.49%)
**Stage_T**	
T1	3 (0.74%)
T2	119 (29.38%)
T3	192 (47.41%)
T4	58 (14.32%)
TX	1 (0.25%)
Missing	32 (7.90%)
**Stage_N**	
N0	235 (58.02%)
N1	46 (11.36%)
N2	75 (18.52%)
N3	8 (1.98%)
NX	36 (8.89%)
Missing	5 (1.23%)
**Stage_M**	
M0	194 (47.90%)
M1	11 (2.72%)
MX	198 (48.89%)
Missing	2 (0.49%)
**Cancer Status**	
Tumor Free	232 (57.28%)
With Tumor	134 (33.09%)
Missing	39 (9.63%)
**Chemotherapy or Immunotherapy**	
Yes	112 (27.65%)
No	287 (70.87%)
Missing	6 (1.48%)

### Differential Analysis and Risk-Score Model Construction

The package “limma” in R with the statistical method of Wilcox Test was used to make the differential analysis of all mRNA expression between tumor group and normal group, and DEG (|log2FC| > 1, *P* < 0.05) was acquired. The volcano and heat maps were plotted by R. Overlaps between immune-related gene and DEG were immune-related DEG. Subsequently, immune-related DEG and clinical information data were merged, data of OS <90 days were deleted, and the univariate Cox proportional hazard regression analysis was performed. Thereafter, 27 immune-related DEG significantly associated with prognosis were obtained, with the package “survival” in R (*P* < 0.01). Overlaps between transcription factor-related genes and DEG were transcription factor-related DEG. The correlation of 27 immune-related DEG with transcription factor (TF) -related DEG was analyzed (|r| > 0.4, *P* < 0.001). The interaction network of TF-related DEG and 27 immune-related DEG was made with the Cytoscape. The “survival” package in R was used to make the multivariate Cox proportional hazard regression based on 27 immune-related DEG. After a number of similar and relevant genes were streamlined, 9-core immune-related DEG were obtained and then Cox regression model was constructed to calculate their respective coefficient (β_i_). An individual's risk score model for each patient was built for predicting prognosis of patients with bladder urothelial carcinoma by including expression level of each optimal prognostic core genes (Exp_i_), which was weighted by their estimated regression coefficients(β_i_) of multivariate Cox regression model. The risk score of each patient was calculate as follows:

$$\begin{array}{l} \text{Risk score }=\;\sum_{\text{i}=1}^{\text{n}}\left(\beta_{\text{i}}\cdot {\text{Exp}}_{\text{i}}\right). \end{array}$$

### Verification of Prognosis Evaluation by Risk Score

By using the median risk score, all patients with urothelial carcinoma were sorted into high-risk group and low-risk group. Kaplan-Meier analysis was utilized to compare the survival between two groups. The survival difference between the low-risk and high-risk group was assessed by the log-rank test. The time-dependent receiver operating characteristic (ROC) curve was performed to identify the exactitude of the Cox regression model by packages “survival” and “survival ROC” in R. According to the risk score and clinical data of each patient, Risk curve, survival state map and heatmap of 9-core immune-related DEG were plotted. Cases with incomplete data were deleted. The “risk score” together with age, gender, grade, stage, “T-stage,” “M-stage,” and “N-stage” was made for univariate and multivariate independent prognosis analysis and the forest map was plotted.

### Correlation Analysis of Clinical Parameters and 9-Core Immune-Related DEG Together With Risk Score

In order to verify the correlation of 9-core immune-related DEG with clinical parameters, cases with incomplete clinical data were deleted, and T-test between Risk score, 9-core immune-related DEG and clinical parameters was analyzed (*P* < 0.05).

## Results

### Identification of Prognostic Immune-Related DEG

**Figure 1:**
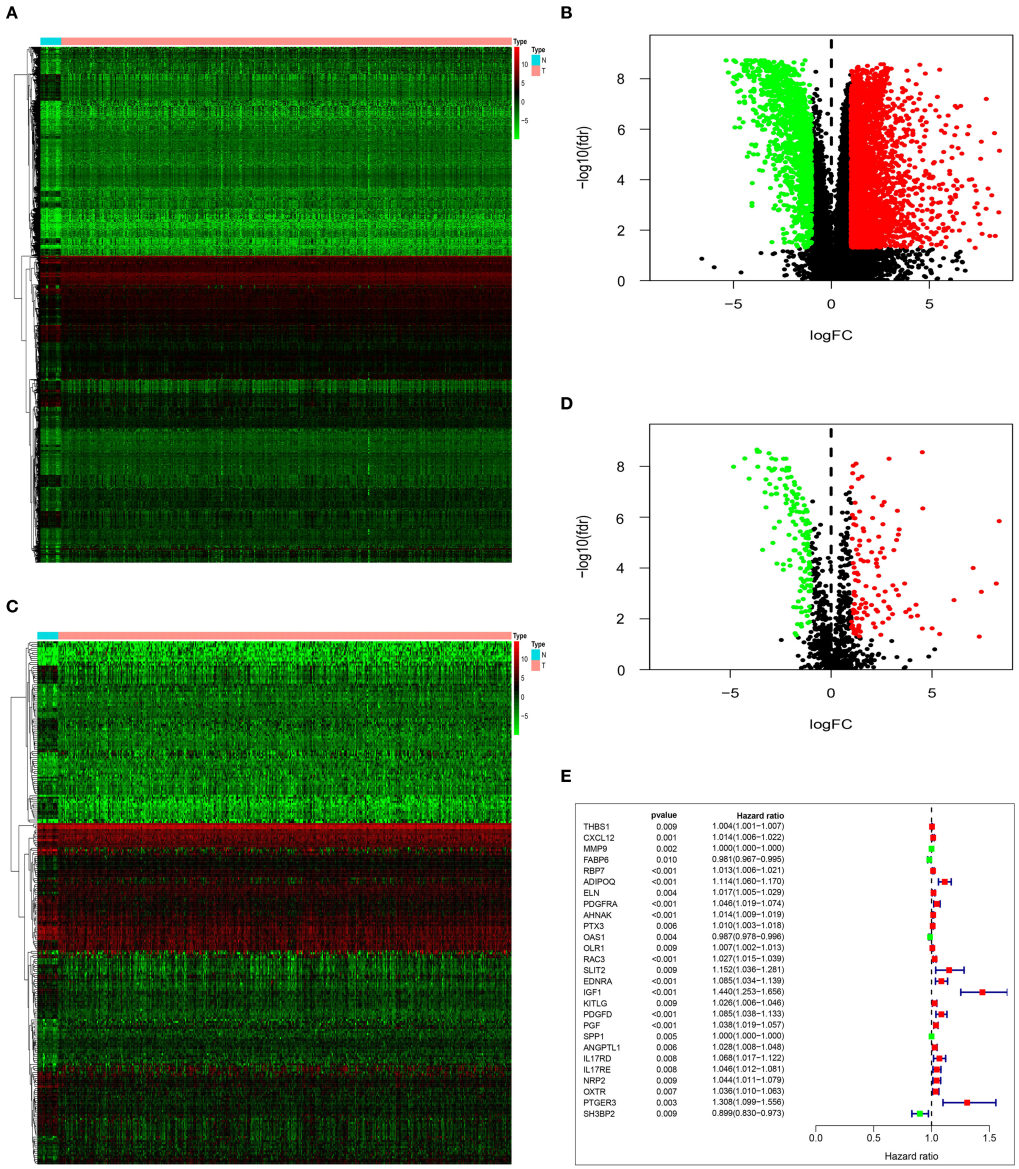
Identification of prognostic immune-related DEG. Heatmap **(A)** and volcano plot **(B)** demonstrating DEG between normal bladder tissues and urothelial carcinoma tissues. Heatmap **(C)** and volcano plot **(D)** indicating immune-related DEG. **(E)** Forest plot of hazard ratios and *P*-value showing the prognostic values of 27 immune-related DEG in univariate Cox proportional hazards regression analysis.

By analyzing the differences between tumor group and normal group, we obtained 4,880 DEG. Based on the differential analysis, the volcanic and heat maps were made (Figures 1A,B). We thereafter crossed these DEG with 2,498 immune-related genes from ImmPort database, and acquired 259 immune-related DEG. The volcanic and heat maps were made similarly (Figures 1C,D). A total of 27 immune-related DEG were found to be significantly associated with the overall survival (OS) of patients with bladder urothelial carcinoma (adjusted *P* < 0.01), and were entered into the candidate pool for further selection (Figure 1E).

### TF-Related Genes Regulating Prognostic Immune-Related DEG

**Figure 2:**
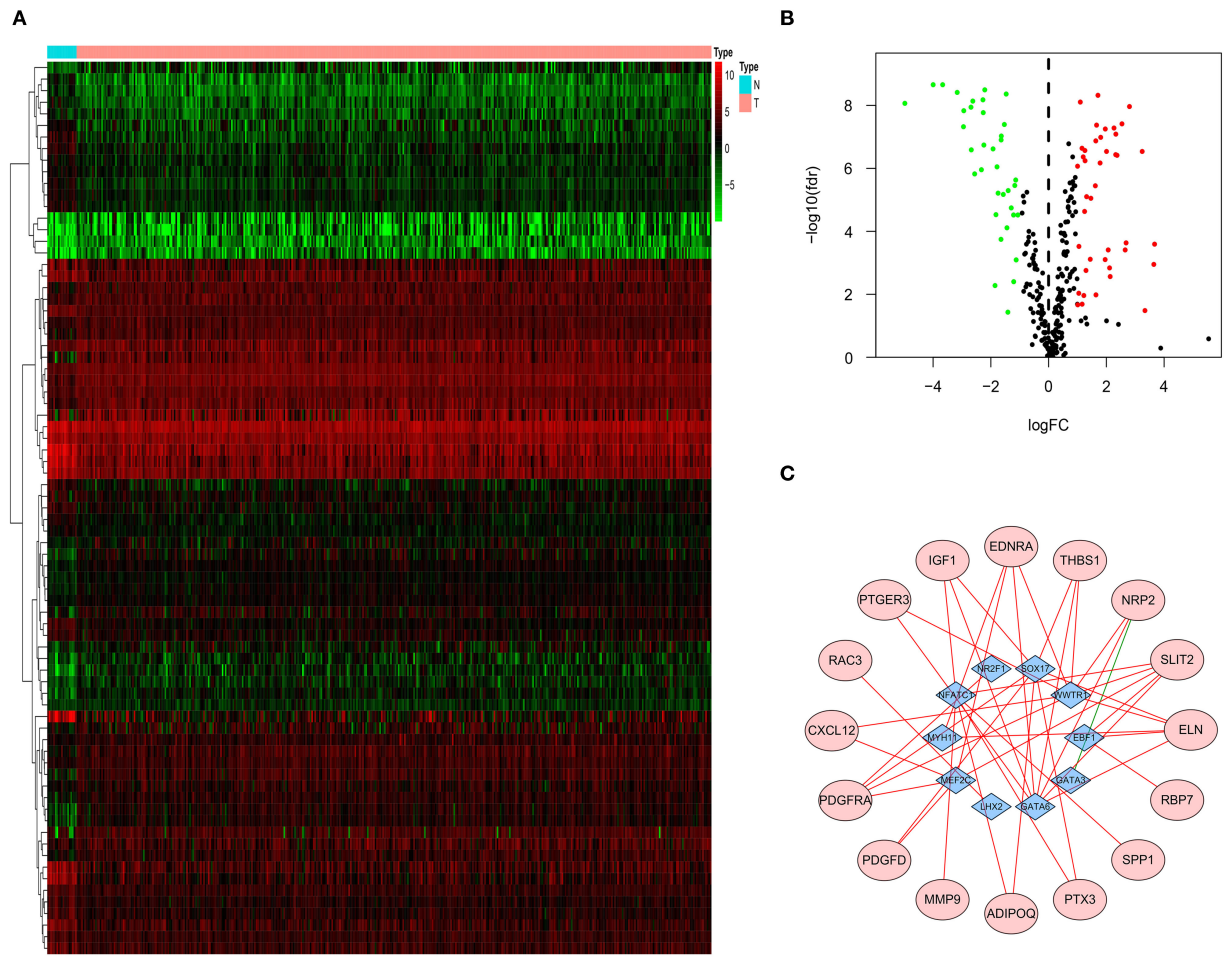
TF-related genes regulating prognostic immune-related DEG. Heatmap **(A)** and volcano plot **(B)** indicating TF-related DEG. **(C)** A gene regulatory network between TF-related DEG and immune-related DEG. The purple ellipses represent the immune-related DEG. The blue diamonds represent TF-related DEG. The red and green solid lines represent positive and negative correlation, respectively.

Three hundred eighteen TF-related genes were downloaded from Critrome. After crossing these TF-related genes with 4,880 DEG, we obtained 77 TF-related DEG (Figures 2A,B). Correlation analysis between 27 immune-related DEG and 77 TF-related DEG was made. There was a correlation between 10 TF-related DEG and 16 immune-related DEG. All of the 16 immune-related DEG were high risk genes. A gene regulatory network by cytoscape was built (Figure 2C).

### Construction of Risk Score Model Using Prognostic Immune-Related DEG

To determine the optimal prognostic immune-related DEG, we adopted multivariate Cox proportional hazards regression using 9-core genes from the pool of 27 candidate prognostic immune-related DEG to evaluate independent prognostic values. In these 9-core DEG, 7 core genes (*MMP9, PDGFRA, AHNAK, OLR1, RAC3, IGF1*, and *PGF*) with positive coefficient of multivariate regression analysis may be risk factors owing to the close association between their high expression and shorter patients' survival, whereas the remaining two core DEG (*OAS1* and *SH3BP2*) tended to be prognostic protective factors and their high expressions were associated with longer survival (Table 2). An individual's risk score model was developed using the regression coefficients of multivariate Cox regression model to weight the expression level of each core DEG. Through this risk score model, we could get the risk score of every case in TCGA database.

**Table 2:** Nine-core immune-related DEG selected to construct risk score model.

**Id**	**coef**	**HR**	**HR.95L**	**HR.95**	***P*-value**
MMP9	0.000325672	1.000325725	1.000135002	1.000516484	0.000815317
PDGFRA	0.029410199	1.02984695	0.994377929	1.066581136	0.100036018
AHNAK	0.013037691	1.013123053	1.008103422	1.018167677	2.68E-07
OAS1	−0.007175947	0.992849739	0.984165276	1.001610835	0.109401278
OLR1	0.00516244	1.005175789	0.9989007	1.011490297	0.106154827
RAC3	0.023591819	1.023872307	1.010181565	1.037748597	0.000592871
IGF1	0.282607117	1.326583867	1.120036922	1.571220307	0.001065029
PGF	0.017645622	1.017802226	0.997880046	1.038122142	0.080196147
SH3BP2	−0.083043743	0.920310889	0.849404006	0.997136965	0.042351158

### Validation of the Risk Score Model

**Figure 3:**
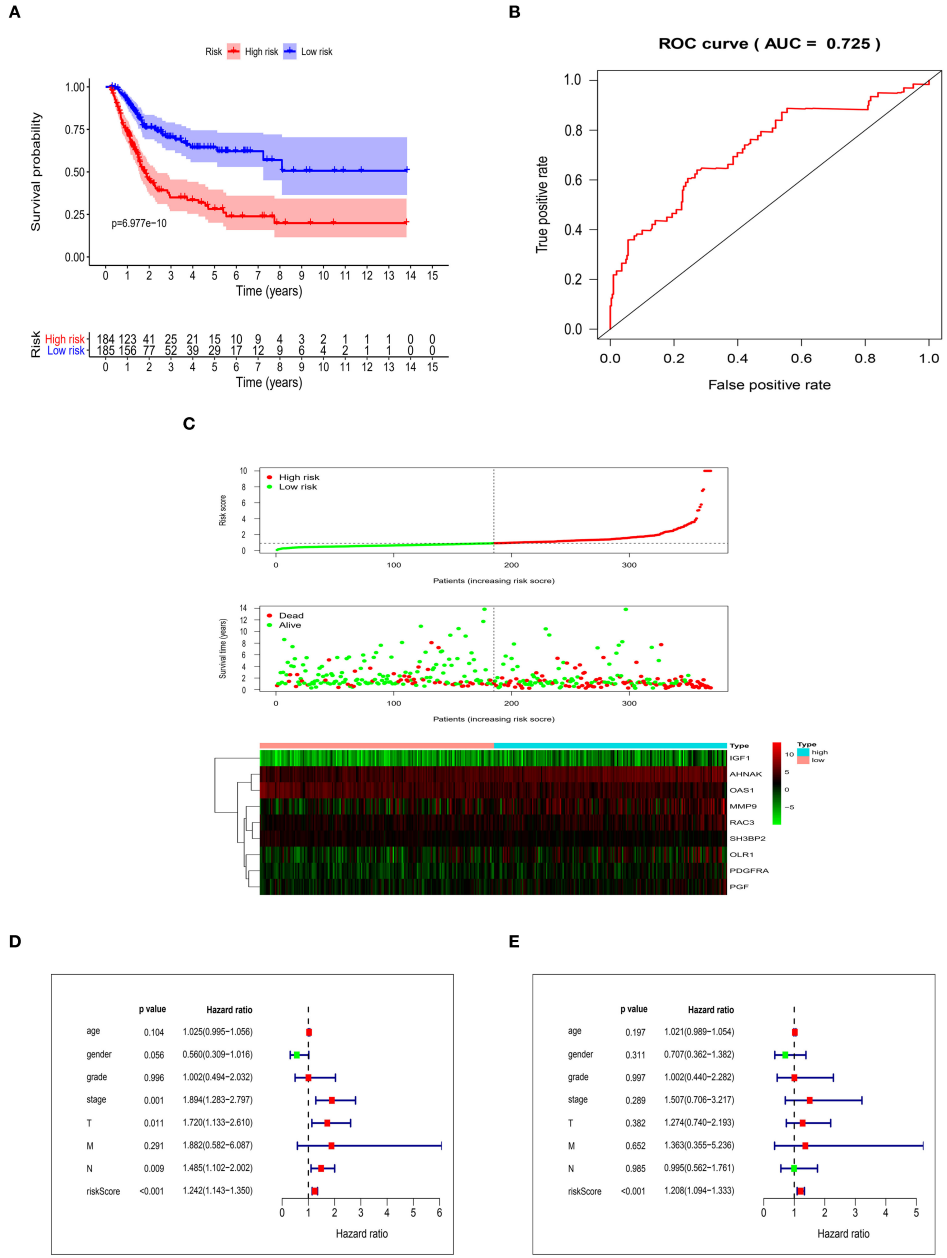
Validation of the risk score model. **(A)** Kaplan-Meier curve showing overall survival of urothelial carcinoma patients with high- and low-risk. **(B)** Analysis of time-dependent ROC curves. **(C)** Risk score distribution, survival status, and prognostic 9-core immune-related DEG patterns of urothelial carcinoma patients with high- and low-risk. Univariate **(D)** and multivariate **(E)** independent prognosis analysis of clinical parameters and risk score.

After getting the risk score of every case, we divided all cases into high-risk group and low-risk group based on median of Risk score. OS was significantly different between two groups (*P* = 6.977e-10). In addition, the 3- and 5-year survival rates of the high-risk group were 35% (95% CI: 26.9–45.5%) and 28.2% (95% CI: 20.1–39.6%), respectively, whereas the corresponding rates in the low-risk group were 70.4% (95% CI: 62.7–79.1%) and 64.4% (95% CI: 55.7–74.5%) (Figure 3A). In order to measure the predictive performance of 9-core immune-related DEG prognostic risk model, we used time-dependent receiver operating characteristic (ROC) curves for 1-year survival. The area under the ROC (AUC) for the 9-core immune-related DEG prognostic model was 0.725 at 1-year of OS (Figure 3B). In the risk curve, we ranked patients by risk score from low to high and found the median. Each patient's OS and survival status were marked in the survival state map. The heatmap revealed expression patterns of prognostic 9-core immune-related DEG (Figure 3C). For patients with high-risk scores, expression of seven risky immune-related DEG was up-regulated while expression of two protective DEG was down-regulated. On the contrary, expression of prognostic immune-related DEG in patients with low-risk scores displayed an opposite pattern to that with high-risk scores. Taking risk score as an independent risk factor, we made univariate and multivariate independent prognosis together with age, gender, grade, stage, “T-stage,” “M-stage,” and “N-stage.” It was found that risk score was the most potential independent factor in both univariate (Figure 3D) and multivariate (Figure 3E) independent prognosis analysis. It was shown that risk score could be used as an important independent factor in evaluating prognosis (*P* < 0.001).

### Correlation of 9-Core Immune-Related DEG as Well as Risk Score With Clinical Data

**Figure 4:**
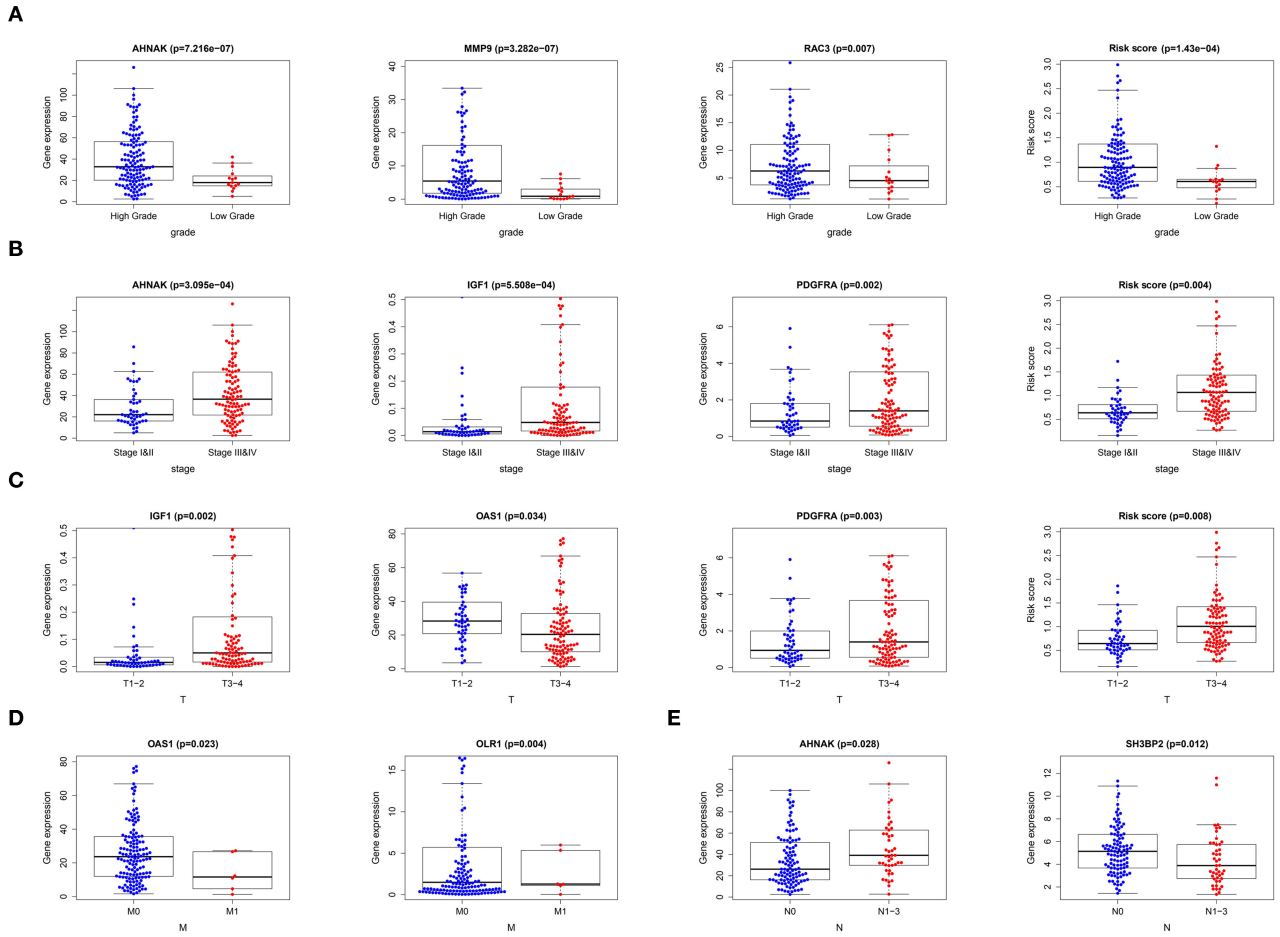
Correlation of 9-core immune-related DEG as well as risk score with clinical data. **(A)** Correlation of gene expression and risk score with tumor grade. **(B)** Correlation of gene expression and risk score with tumor stage. **(C)** Correlation of gene expression and risk score with tumor “T-stage.” **(D)** Correlation of gene expression with tumor “M-stage.” **(E)** Correlation of gene expression with tumor “N-stage”.

After merging 9-core immune-related DEG expression and clinical data and excluding the patients with incomplete information, we made T-test between 9-core immune-related DEG expression as well as risk score and age (≤65/>65), gender (male/female), grade (low/high grade), stage (stage I-II/III-IV), “T-stage (T1-2/T3-4),” “N-stage (N0/N1-3),” “M-stage (M0/M1).” Several core genes were related to clinical parameters (Figure 4) (Table 3). *MMP9* and *RAC3* were highly expressed in high grade. *PDGFRA, IGF1*, and *AHNAK* were highly expressed in high grade, stage III-IV and T3-4 stage. On the contrary, OAS1 was highly expressed in ≤ 65 years old group, male, low grade, stage I-II, T1-2 stage and M0, and SH3BP2 was highly expressed in N0. Finally, risk score was significantly high in high grade, stage I-II and T3-4 stage. These data indicated that risk score together with *MMP9, PDGFRA, AHNAK, RAC3, IGF1* were risk factors while *OAS1* and *SH3BP2* were protective factors.

**Table 3:** Correlation of 9-core immune-related DEG as well as risk score with clinical data.

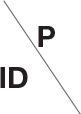	**Age (≤65/>65)**	**Gender (male/female)**	**Grade (low/high)**	**Stage (I–II/III–IV)**	**T (T1–2/T3–4)**	**M (M0/M1)**	**N (N0/N1–3)**
MMP9	0.938	0.513	3.28E-07	0.105	0.048	0.322	0.573
PDGFRA	0.591	0.401	0.004	0.002	0.003	0.66	0.232
AHNAK	0.449	0.157	7.22E-07	3.10E-04	1.29E-04	0.382	0.028
OAS1	0.034	0.013	0.046	0.034	0.034	0.023	0.079
OLR1	0.448	0.981	0.164	0.277	0.292	0.004	0.717
RAC3	0.703	0.305	0.007	0.588	0.947	0.309	0.093
IGF1	0.925	0.397	0.004	5.51E-04	0.002	0.373	0.279
PGF	0.732	0.529	0.357	0.38	0.788	0.431	0.039
SH3BP2	0.427	0.832	1	0.077	0.371	0.908	0.012
Risk score	0.774	0.101	1.43E-04	0.004	0.008	0.776	0.175

## Discussion

Human tumors are characterized by infiltration of immune cells such as lymphocytes, macrophages, dendritic cells (DC), granulocytes and mast cells. These cells are located in different areas of a tumor. However, density and location of immune cells vary greatly in different tumors with the same cancer type, indicating that the distinct immune pattern may affect clinical outcome. A large body of research has revealed that levels of lymphocyte infiltration was associated with prognosis in a series of human cancers (14). Although the effect of CD4^+^ T cells on clinical outcome was debatable, high infiltration of CD8^+^ cytotoxic T cells and memory T cells were clearly shown with a longer survival, and these patients demonstrated an increased expression of T-helper 1 and cytotoxicity-related genes (15). Moreover, an immune scoring was proposed based on the type, density and location of lymphocyte infiltrates as a novel prognostic factor (16–18).

In urothelial carcinoma, it was shown that patients with advanced disease (pT2, pT3, or pT4) who have higher numbers of CD8^+^ T cells within the tumor had longer survival than did patients with similar stage and fewer CD8^+^ T cells (19). However, study by Wang et al. observed that high stromal CD8^+^ T cells were associated with poorer OS, and PD-L1^+^ immune cells were an independent prognostic factor for OS and recurrence-free survival in patients with bladder urothelial carcinoma (20). Intriguingly, this study showed that PD-L1^+^ tumor cells were also associated with unfavorable survival. Recently, an 18-gene signature that molecularly defines urothelial cellular differentiation was identified with prognostic value (21).

In the present study, we identified 27 immune-related DEG significantly associated with OS of patients with bladder urothelial carcinoma. Moreover, we constructed a TF-mediated network to uncover TFs that could regulate expression of 27 immune-related DEG. We further developed a 9-core DEG signature which could be adopted to classify patients into the high-risk group and low-risk group with significantly different OS. The 9-core DEG included protective *OAS1* and *SH3BP2* and risky *MMP9, PDGFRA, AHNAK, OLR1, RAC3, IGF1*, and *PGF*. We verified the prognostic value of the 9-core DEG signature. Results suggested that the 9-core DEG signature had good repeatability and stability in predicting prognosis of patients with bladder urothelial carcinoma. The 9-core DEG signature was independent on traditional clinical risk factors and molecular features.

MMP9 is a member of matrix metalloproteinase family, promoting cancer invasion, metastasis and angiogenesis. Its expression was correlated with tumor grade, invasiveness and unfavorable prognosis of patients with bladder urothelial carcinoma (22, 23). AHNAK is a giant protein which exerts diverse functions in different human cancers. It was shown that AHNAK was preferentially expressed in the nucleus of urothelial carcinoma cell (24). The insulin-like growth factor-1 (IGF1) pathway is associated with growth, metastasis and clinical outcome of various cancers. Urothelial overexpression of IGF1 increases susceptibility to p-cresidine-induced mouse bladder cancer, especially transitional cell carcinoma (25). Furthermore, patients with bladder cancer had higher plasma levels of IGF-1, which were associated with an increased risk of bladder cancer (26). Until now, the roles of PDGFRA, OLR1, RAC3, PDF, OAS1, and SH3BP2 in bladder urothelial carcinoma remain largely unexplored.

Some recent investigations based on databases such as TCGA and GEO are related to our findings. Qiu et al. identified seven prognostic immune-related genes in patients with bladder cancer, including RBP7, PDGFRA, AHNAK, RAC3, EDNRA, OAS1, and SH3BP2 (27). Consistent with our study, PDGFRA, AHNAK and RAC3 were risk factors, whereas OAS1 and SH3BP2 were protective factors. Another study revealed that DEG between bladder cancers and normal tissues were enriched in PDGF signaling and immune-related signalings (28). Moreover, PDGF signaling contributed to immune microenvironment and unfavorable prognosis in an analysis of multiple tumors (29). Kudryavtseva et al. reported that RAC3 was associated with poor prognosis of patients with prostate cancer (30). High AHNAK in pancreatic ductal adenocarcinoma (PDAC) was associated with short survival (31); however, in melanoma low AHNAK was associated with poor prognosis, suggesting a tumor-specific role of AHNAK in prognosis (32).

In conclusion, we for the first time identified and validated a 9-core immune-related genes signature with independent prognostic significance for patients with bladder urothelial carcinoma. These 9-core immune-related genes can be adopted alone or maybe better in combination, to predict clinical outcome of urothelial carcinoma. However, our study is limited because it is retrospective. For better clinical application, the capacity of our signature for prognostic evaluation should be tested. Meanwhile, functional and mechanistical investigations on the 9-core genes in urothelial carcinoma should be further performed.

## Data Availability Statement

Publicly available datasets were analyzed in this study, these can be found in The Cancer Genome Atlas (https://portal.gdc.cancer.gov/).

## Author Contributions

LN and CZ proposed the study concept, design, and drafted the manuscript. YB and YS collected, analyzed, and interpreted the data. WW, ZW, and LY participated in revising the manuscript. All authors contributed to the article and approved the submitted version.

## Conflict of Interest

LY was employed by the company Liaoning Branch of China Telecom. The remaining authors declare that the research was conducted in the absence of any commercial or financial relationships that could be construed as a potential conflict of interest.
